# On Variations of Pitch in Percussion and Respiratory Sounds, and Their Application to Physical Diagnosis

**Published:** 1852-12

**Authors:** Austin Flint

**Affiliations:** Buffalo, New York


### SELECTIONS

FROM

### FOREIGN AND HOME MEDICAL JOURNALS.

---

*On Variations of Pitch in Percussion and Respiratory Sounds, and their application to Physical Diagnosis.* By Austin Flint, M. D., of Buffalo, New York.—Very litttle attention has hitherto been paid to variations in the pitch of sounds heard in the practice of percussion and pulmonary auscultation. The sibilant and sonorous râles of bronchitis, it is true, are distinguished from each other chiefly by a contrast in pitch, but, as respects the remainder of the physical signs pertaining to pulmonary disease, they appear not to have been much studied in this aspect, and even the facts that have arrested notice do not seem to have been applied, save in a very limited degree, to physical diagnosis. By most writers on physical exploration, pitch modifications, except* in the sibilant and sonorous râles, are not recognized, no allusion whatever being made to them. In the second edition of the able and comprehensive work of Dr. Walshe, of London, recently republished in this country, the subject is noticed more distinctly than by any other author with whose writings I am acquainted. Dr. Walshe enumerates among the elements involved in the different modifications

---

* The vocal sign *aegophony* should perhaps also be excepted.

of respiratory sounds in health and disease, variations in pitch; he also mentions several important facts with respect to these variations. But he apparently loses sight of their practical applications, making no reference to them in connection with the diagnosis of individual thoracic diseases. Barth and Roger state, as briefly as possible, the fact that the bronchial respiration is higher in pitch than the cavernous. But, in general, as just remarked, nothing is to be found relating to this subject in standard treatises* on percussion and auscultation.

What facts are disclosed by the study of percussion and respiratory sounds, in health and disease, with reference to variations and pitch? How far are these facts available in diagnosis? The latter question is much the more important of the two; or, rather, the importance of the first question depends mainly upon that which belongs to the second. The subject of physical exploration has already suffered from over refinement. To be practically available in the hands of all intelligent practitioners, the art must be simplified as much as possible. New distinctions, unless obviously tending to enlarge or facilitate our means of diagnosis by physical signs, were they not only true, but ever so interesting to those whose attention is specially directed to this department of medicine, would be of questionable utility, so far as they serve to render the pursuit mere complicated and difficult. In proposing, therefore, to submit the results of my observations and experience, thus far, in answer to the foregoing inquiries, I am influenced by the belief that, considered in this point of view, the physical signs of pulmonary affections admit of being enlarged in their application to diagnosis, and rendered more readily available for practical purposes. A single additional remark by way of introduction; the

---

* In connection with this statement, it is proper to give the bibliography to which it applies. The works consulted are as follows:—

Laennec, edited by Forbes; Walshe on the Heart and Lungs; Hughes's Physical Diagnosis of the Lungs and Heart; Barth and Roger's Practical Treatise on Auscultation; Gerhard on Disease of the Chest; Prize Dissertations on Physical Exploration, by Drs. Holmes, Bell, and Haxall; Blackiston on Diseases of the Chest; Latham on Auscultation and Semeiology; Swett on Diseases of the Chest, edition of 1852; Bowditch's Young Stethoscopist; Lawson's Lectures, in the Western Lancet.

pitch modifications of sound, as before intimated, opening a field of study in physical exploration as yet but little cultivated, and to which, so far as relates especially to auscultation, my attention has but recently been directed, propriety and prudence dictate, not only caution in making deductions from a number of data somewhat limited, but a certain amount of distrust in a kind of observation in which the liability to error cannot be at once fully estimated. In view of these considerations, the conclusions which I shall present are advanced as propositions to be confirmed by further researches, the object of this paper being, in a great measure, to invite the investigations of others in the same direction.

The subject naturally resolves itself into the study of—First, *percnssion* sounds; and, second, the pulmonary sounds disclosed by *auscultation.*

SECTION 1. *On attention to Pitch of Sound in the practice of Percussion.*—It is hardly necessary to gather recorded data with reference to the variations of pitch of *percussion* sounds, since observations can be so easily made, and repeated, to any extent, by every one. I shall therefore give my experience under this head briefly, and in a general way, *i. e.* without citing particular cases. The use of the term *pitch*, as applied to percussion sounds, may be questioned, and questionable. Whether it be correctly applicable, in a musical sense, to these sounds, or to those of auscultation, I shall not stop to inquire. It suffices if the idea it conveys be intelligible, and if it denote an appreciable distinction. A percussion sound elicited from the chest, if not entirely flat, ha a certain amount of resonance. This resonance may differ in degree; in other words, the sound is more or less clear or dull. A peculiar quality of tone arises from the fact that the chest contains air in vesicles, and hence the sound may be distinguished as the *vesicular resonance.* Percussion sounds present occasionally, deviations from this vesicular quality; an example the most familiar being the change occasioned by the presence of air between the pleural surfaces, or in a large excavation, constituting what is known as tympanitic resonance. Now, in addition to these classes of variations, the resonance on percussion in different parts of the chest, in morbid conditions, present a disparity analogous to, if not identical with that which constitutes the distinction called *high* or *low* in comparing musical tones—in other words, a disparity in pitch. This kind of disparity is recognized by directing attention to, and comparing the sounds elicited from different regions, just as is done with reference to musical notes, in determining whether they are relatively higher or lower in key. The faculty of distinguishing musical discords, in other words a musical ear as it is called, doubtless assists this discrimination, and it is probably true that the ability to detect a slight variation will correspond with the delicacy with which deviations from the harmony of musical tones are appreciated.

What pathological significance belongs to the fact of a variation in the pitch of resonance? The practical importance of the subject depends, of course, on the answer to this question. The question leads to the enunciation of the following law: An elevation of pitch always accompanies diminution of resonance in consequence of pulmonary consolidation. In other words, dullness of resonance is never present without the pitch being raised. This proposition is to be verified by observations which may be readily made. Cases of tuberculous deposit, with marked disparity of resonance at the summit of the chest on one side will furnish examples, and such cases are sufficiently numerous. But the law may be tested in the healthy subject. The præcordial region presents well-marked dullness compared with the corresponding region on the right side. The percussion sound in the former situation will answer as a type of resonance, modified by an increased proportion of solid material incident to tuberculous or other disease

A practical advantage of attention to pitch, in employing percussion, consists, thus, in its confirming the existence of dullness. It furnishes additional evidence thereof, and adds positiveness to the conclusion, when the diminution of resonance might not be with certainty determinable. To the musical ear, more especially if skilled in discriminating musical tones, a disparity in pitch is more quickly as well as more clearly distinguished. It is far easier to appreciate a contrast in this point of view, than to determine a slight or moderate preponderance of resonance in the percussion sound on one side of the chest. I have frequently illustrated this in teaching physical exploration. A person just essaying to distinguish relative dullness of resonance on percussion, often fails in its recognition, even when it is pretty strongly marked. He is unable to perceive a difference which is sufficiently apparent to the practical percussor. Under these circumstances I have frequently inquired if the learner were able to sing, or play on any musical instrument. If he replied in the affirmative, I requested him to compare the sounds on the two sides of the chest, as if they were musical notes, with reference to pitch, and the disparity then became immediately manifest. If I am misled with respect to the assistance to be derived in this way, by the results of my own experience, the error involves a kind of self deception which it is very easy to impart, for many to whom I have pointed out this method of determining dullness, have assured me that they have found it of great utility in practice. By directing attention to the pitch, an intelligent student, with some musical powers and cultivation, will become an expert in appreciating a slight dullness sooner than he can attain to proficiency in the manipulating tact of percussion.

Another practical advantage, certainly equal to, if not greater than the foregoing, is derived from the fact that a distinct disparity of pitch may be apparent, in some instances, when a disparity in the amount of resonance is inappreciable. The correctness of this statement my observations lead me not to doubt. Others, however, are to be satisfied by the evidence of their own perceptions. This fact is stated by Dr. Bowditch in his work entitled the *Young Stethoscopist*, and I do not recollect meeting with a similar statement by any other writer. He says, "A *difference of note or of pitch* between two corresponding parts is not uncommon, when there is no real flatness in either. It occurs in cases in which the lung is not by any means impervious to air. Sometimes in the early stages of phthisis and in pneumonia in its early or latest stages;" (page 60, second edition.) In another place he remarks: "Any degree of dullness, even the slightest *difference of note or of pitch, if confined to the upper part* of the chest, between the portions equidistant from the spine or sternum, augments my suspicion of the existence of tubercles;" (page 88, ibid.) To measure the exact amount of mere resonance, so as to make an accurate comparison of two sounds that differ but slightly in this respect, it must be evident on a little reflection, is not easy. The truth probably is that the difference in pitch is perceived when a slight disparity exists which is ordinarily called dullness. And this leads me here to remark, it is not to be supposed that practical chest explorers have not been accustomed to be guided by pitch modifications of sound. Variations in this respect have been perceived without recognition, if this antithesis be allowable. The disparity has been apparent, but attributed solely to diminished resonance or dullness. This remark will be found to apply equally to respiratory or to percussion sounds. This, however, does not prove that the practice of physical exploration will not be materially aided by directing the attention to the variations in pitch *as such.*

A contrast in percussion sounds in many of the diseases of the chest is sufficiently obvious, requiring no special delicacy of discrimination. This is the case in pleuritis with effusion, in the second stage of pneumonitis, and in phthisis when the tubercelous deposit is abundant. Attention to pitch, under these circumstances, may be said, with truth, not to be of importance. A flat, and notably dull sound are readily enongh discovered. It is in connection with the diagnosis of the early stage of tuberculosis that the point under consideration is particularly important. Of the importance of the diagnosis of the disease in this stage it is not necessary to speak. In view of the employment of means to arrest the further progress of tuberculization, and the fair prospect, in many instances, of effecting that object, there is perhaps no end in practical medicine more desirable than to determine positively the presence of tubercle before the disease has made much progress. Any addition to our means of giving precision to the diagnosis of incipient phthisis is a valuable contribution, not only to our science, but to our art, inasmuch as the prospect of saving or prolonging life is greater in proportion as the affection is earlier recognized.

A disparity at the summit of the chest, however slight is a sign to which great weight should be attached in deciding on the presence of tubercle. Practical auscultators generally will concur in the statement above quoted from the treatise by Dr. Bowditch, relative to this point. If, therefore, by attention to the pitch of resonance, we are better able to appreciate a slight shade of difference, or to recognize its existence with greater certainty, there can be no question concerning the service thereby rendered to physical exploration.

It is hardly necessary to say, that the usefulness of the means of early diagnosis in phthisis is to be regarded in both a positive and negative aspect. In other words, it is quite as important to be able to exclude tuberculosis, by the absence of physical signs, as to determine its existence by the presence of these signs, and, hence, improvements in physical exploration have practical relations of equal value, in either direction.

Another advantage to be derived from attention to pitch consists in the facility of developing a disparity in situations in which but little resonance can be elicited, owing to the intervention of bone, muscle, and fat between the integument and walls of the chest. A late writer on diseases of the chest says, that the mammary region in females "is of no value in percussion."* The same author farther states that, "posteriorly, no advantages can be derived from percussion; the muscles are too thick about the shoulders to admit of a decided advantage even in cases in which the pulmonary condensation is much greater than in incipient phthisis" These statements are to me surprising. A disparity of pitch is frequently very apparent in the scapular region, both above and below the spine, in cases of tuberculosis, and I have met with it here when it was not marked in the infra-clavicular region. I have demonstrated this fact repeatedly during the short period that has elapsed since the publication of the work from which the above extracts are taken. To dispense with the information from percussion sounds over the scapulæ would considerably impair the diagnostic resources in incipient phthisis. The thickness of the muscular coating in other parts of the chest, and even the

---

* A Treatise on Diseases of the Chest, by John A. Swett, M. D., 1852, pp. 17 and 258.

mamma of the female, do not prevent a comparison of sounds with reference to pitch ; but, for reasons obvious enough to the pathologist, the comparison in these situations is less important in diognosis, save in diseases in which the coarsest exploration would suffice to develop flatness, as in pneumonitis and pleurisy.

Another advantage relates to the diagnosis of heart diseases. Percussing from the clavicle, the acromial angle, and the axilla, in a direction toward the site of the heart, the points at which there is a distinct modification of pitch will mark the limits of the organ in these directions, while the point of impulse, if perceptible, will give the lower boundary. This method, thus, will be found useful in determining the degree of enlargement in hypertrophy and dilatation, and the amount of effusion in pericarditis.

The significance of variations in pitch, as of other points of disparity, in the diagnosis of thoracic diseases, of course, is predicated on the equality of both sides of the chest, in this respect, in health. Does this equality exist? I cannot answer this question quite so explicitly as I could desire. Pitch modifications will necessarily result from causes which at the same time occasion obvious dullness, such as spinal curvatures, former pleurisies, arrested tuberculous deposit, &c. These causes, and others, moreover, may occasion, in some instances, a disparity in pitch, when the changes are not sufficient to produce obvious dullness. Comparison in pitch affording a more delicate means of discovering a slight deviation from the symmetry of the two sides, it is not improbable that healthy chests may be less uniformly equal in this particular, than with respect to a marked diminution of resonance on one side. The examination of a large number of individuals presumed to be free from pulmonary disease would alone afford the data for answering the above question positively. I have frequently examined chests, at the summit more particularly, with reference to this point, but without recording the results. Within a few days I have noted observations in twenty-two persons. In all save *three*, the pitch was equal in the infra-clavicular region, to which attention was more particularly directed. In *one*, there was elevation of pitch on the right side, apparently owing to the greater development of the muscles on this side. In another, the same disparity existed, the same explanation not being so obvious. There was no reason to suspect disease of the lungs in this latter instance. The patient was a young female in perfect health. In the third instance the percussion sounds were clear and equal at the summit anteriorly, but over the right scapula there existed evident dullness compared with the sound over the left scapula. This person was a mechanic, and the muscular mass in the interscapular space was notably thicker on the right side. It is undoubtedly desirable that examinations of this kind should be multiplied.

In practicing percussion with a view to *pitch*, observance of the rules which are familiar to every practical auscultator is peculiarly important ; I allude to the position of the patient, care to strike successively on the two sides at corresponding points, and with an equal force, &c. Tact in elicting a loud distinct sound is desirable. A smart stroke is frequently more effective for this object than a light feeble blow, not, however, using sufficient force to occasion pain. The only pleximeter and precussor with which I have any practical acquaintance are the fingers. It has often occured to me that other instruments might in some instances be more satisfactory, but I have nothing to say on this topic from my own experience.

SECTION 2. *On attention to the pitch of sound in the practice of auscultation.*—My attention has been directed to the variations in the pitch of respiratory sounds more recently, than to those belonging to percussion.—It is for a few months only that I have made the former objects of study During this time, as leisure and opportunities have permitted, I have been accustomed to note observations on the different cases of thoracic diseases coming under my notice ; and the views which I shall submit in this section will consist of deductions from the data thus collected. Considering the novelty of the subject, as well as its importance, I have thought it best to present, not only the conclusions based on what data I have gathered, but a transcript of the data themselves. As an appendix to this section, therefore, I shall give a brief synopsis of the observations which have been made in cases of disease, amounting to over forty in number. A larger collection of data for generalization is undoubtedly to be desired; but it is to be borne in mind that the aim of this memoir is by no means to exhaust, but rather to open the subject, as one claiming attention, with a view to its practical application to physical diagnosis. I would here repeat that what I shall advance as the results of observations made up to this time, I wish to be considered in the light of propositions to be confirmed, enlarged, and perhaps corrected by continued investigations. As already intimated, to be able to estimate fully the liability to error of observation in a new field of study like this, may require the fruits of a longer experience. I am led to make this remark the more, in consequence of being sensible that a facility in distinguishing pitch variations in the respiratory sounds is much increased by practice. After what I have said, it is but justice to myself to add that, in making the observations I have noted, I have spared no effort to render them reliable.

The point of departure for the study of pitch modifications, as of other physical signs of disease, is the examination of the chest in health. The first inquiry, therefore, in presenting this branch of the subject, will be, *what variations in pitch belong to healthy respiration?* To this question I will devote a distinct division of s section.

*Variations in the Pitch of Sounds in Healthy Respiration.*—With a view to the study of the variations to be found in a healthy chest, I have noted the results of physical explorations, more or less complete, of twenty-seven individuals, presumed to be entirely free from any thoracic disease. This number of observations, although too few to settle the numerical ratio of the occurrence of particular phenomena, will probably suffice for the present object, which is merely to determine some general principles to serve as the basis of the study of morbid deviations.

Of these twenty-seven individuals twenty-one were males, and six females. The ages were various, all, with a single exception, being above childhood, and none being advanced in life. The majority were young persons from twenty to thirty years of age.

The normal respiratory sounds are resolvable into three divisions, according to the parts of pulmonary apparatus, whence they emanate, viz: the trachea, the bronchi, and the vesicles. Named from these their anatomical relations, they are the *tracheal*, the *bronchial*, and the *vesicular*. The character of pitch belonging to the sounds produced in these three situations may be considered under three distinct heads.

1. *Tracheal Sounds.*—On placing the stethoscope over the trachea, the respiratory sounds are found to be notably high in pitch. The development, that is to say the loudness of the sounds, in ordinary respiration, varies considerably in different persons. In some persons they are quite intense, in others feeble, and even indistinct until the persons are requested to breathe forcibly, when they become much increased. The relative altitude of pitch is immediately perceived on comparing the tracheal sounds with the vesicular respiration heard on listening over the chest. Two sounds are heard uniformly in this situation, viz: the sounds of inspiration and expiration. An interval occurs between these two sounds. The inspiratoty is relatively shorter than the expiratory sound. The sound of expiration is higher in pitch than that of inspiration. These results have been uniform in all the observations that I have made.

2. *Bronchial Sounds.*—Explorations for the bronchial respiration were made anteriorly near the clavicula-sternal junction, and, in a smaller portion of the cases, in the interscapular space posteriorly. Of twenty-three examinations in the former of these situations, the bronchial sound was appreciable in twenty, and not discoverable in the remaining three instances. Of fourteen examinations in the latter situation, *i. e.*, the interscapular space, it was appreciable in all but one instance. As respects the two situations, in some cases, the sounds were more developed posteriorly and anteriorly, and in other cases, the reverse. There was considerable difference in different cases in the degree of development, or intensity of the sounds. In several, no sound was discernible during ordinary respiration, but it became apparent on increasing the force and quickness of the respiratory movements.

The pitch of the bronchial sounds is high, probably no much below that of the tracheal sounds, but it did not occur to me to compare the two with reference to pitch, until I began to write on the subject. The point has not much importance. The elevation of the pitch of bronchial, compared with the vesicular murmur, is to be borne in mind. A comparison of the former, as heard in the interscapular space, and near the sternum anteriorly, with the latter as heard over other parts of the chest, showed, in every instance, a distinct and notable disparity.

Other interesting points of distinction pertain to the *bronchial sounds.*

Recollecting the relations, in size and length, of the two primary bronchi to each other, the inquiry arises if the bronchial respiration heard in corresponding situations on both sides of the chest, before and behind, is uniformly equal in intensity and pitch? With respect to intensity or loudness, of *thirteen* examinations it was thought to be somewhat more developed on the left side in four cases, on the right side in five, and in four cases no difference in this particular was apparent. As regards pitch, the result was different. Of twenty examinations the pitch was noted to have been distinctly higher on the right side in fifteen, no difference being appreciable in the remaining five cases. This disparity in pitch between the two sides was not observed in all instances, both anteriorly and posteriorly. Excluding seven instances in which it is merely noted that the pitch was higher on the right side, without specifying whether before or behind, the notations in the remainder of the cases are as follows: 1. No disparity in front, but notably higher on the right side behind. 2. Higher on the right side before and behind. 3. The same. 4 The same. 5. Higher on the right behind, where the bronchial sound is alone heard. 6. Slightly higher on the right side in front, no difference being appreciable behind. 7. Higher on the right side before and behind. 8. The same.

The existence of disparity in the bronchial sounds of the right and left side, attributable to the difference in size and length of the bronchial divisions of the trachea, has been peinted out by Dr. Gerhard, of Philadelphia, but the fact has not been recognized by all auscultators. Dr. Stokes, on the contrary, has taught that a disparity frequently exists, but that the bronchial character is apt to be more apparent on the left than on the right side. Dr. Gerhard speaks of the respiration being more blowing or tubular on the right side. If my observations are correct, both Dr. Gerhard and Dr. Stokes may be right, the discrepancy arising from confounding different elements which enter into the bronchial respiration. It is occasionally more developed, that is to say, louder on the left than on the right side. So far Dr. Stokes is correct, but in a very large majority of persons the pitch is higher on the right side, while this relative elevation never obtains on the left; and, as the pitch is one of the most prominent of the elements of the bronchial respiration, the foregoing results accord with the fact pointed out by Dr. Gerhard. The observations of these two distinguished writers are thus apparently not really inconsistent with each other.

In degree, or loudness, the normal bronchial respiration presents in different individuals great variations. In this point of view it has no uniformity. It is never intense, and, in some persons, is quite faint, requiring a forcible respiration to develop it sufficiently to study its characters; and it is not appreciable in all persons.

In a certain proportion of the cases in which the normal bronchial respiration is heard, not uniformly, a sound of expiration is appreciable. Of thirteen instances in which the inspiratory sound was present, a sound of expiration also existed, the latter being absent in four. The facts with respect to these variations contained in the few observations I have noted are as follows: Of the above thirteen instances, in six the sound of expiration was perceived only on the right side, either in front or behind, and in seven it was appreciable behind, and not in front.

The sound of expiration appeared to be higher in pitch than the sound of inspiration, in every instance in which attention was directed to this point. This fact is noted in nine observations, an exception thereto not being noted in any. Thus, in this trait, the normal bronchial respiration resembles the tracheal.

In every instance in which attention was directed to the succession of the sounds of inspiration and expiration, an interval between was observed. This feature belongs to the bronchial, in common with the tracheal respiration.

It will thus be noticed that, in the more important of the elements of the tracheal and bronchial sounds, they are similar. They both want a distinctive quality which will be seen to characterize the vesicular respiration. They are both high in pitch, in this respect probably not differing much from each other. The inspiratory sound in both is short. The expiration in each is higher in pitch than the inspiration; the difference with respect to this point between the tracheal and the bronchial respiration, being that in the latter an expiratory sound is heard in a certain proportion of cases only, while it is uniformly heard in the former. In each, an interval occurs between the sounds of inspiration and expiration. The characters distinguishing the two kinds of respiration consist in the greater intensity of the tracheal sounds, the uniformity with which they are heard in different persons, and the constant presence of an expiratory sound.

3. *Vesicular Respiration.*—The vesicular respiration, as is well known, differs from the tracheal and bronchial in having a peculiar quality, not capable of being very definitely expressed by language, but which is readily enough appreciated by the practiced ear. The terms *breezy* and *expansive*, perhaps, approach as near a definition as can be done by words. This quality of sound is *sui generis*, and familiarity with it is very necessary to the practical auscultator. The peculiarity alluded to may be characterized as, *par excellence*, the *vesicular quality*. I shall have occasion to refer to it hereafter by this title.

In what other particulars does the vesicular respiration differ from the two varieties already noticed?

The difference in pitch is striking. The pitch is uniformly and notably lower than that of the tracheal or bronchial respiration. This was true of all the cases examined with reference to healthy variations. As a point of difference, it is one to which, in connection with the subject of this memoir, special attention is desired.

An expiratory sound is appreciable in a less number of instances than is the case with the bronchial respiration. Of nineteen examinations with reference to this point, the expiration was heard in nine. The pitch of the expiratory sound, when it is heard, compared with that of the inspiration, is another point of special interest in the present inquiries. Of eight observations in which the facts relating to this comparison were noted, the pitch of the expiration was lower in all but two instances. In these two instances, the pitch of expiration was higher in the right infra-clavicular region towards the sternum, no expiratory sound being appreciable, in either instance, except in the situation first mentioned. So far as these examinations go, then, the rule is, that the sound of expiration in vesicular respiration is lower in pitch than the sound of inspiration, with occasional exceptions* at the summit of the chest on the right side towards the sternum. This rule, without the exceptions, is stated by Dr. Walshe in the last edition of his work on the heart and lungs.

Aside from the foregoing differances, the duration of the inspiratory sound, in the vesicular respiration, is longer than in the tracheal or bronchial. The expiratory sound, on the other hand, is as notably shorter; and when a sound of expiration is appreciable, it is nearly or quite continuous with the sound of inspiration. The latter statement I give on the authority of others, and my own unrecorded experience, not having taken pains to note the facts relating to the point in healthy observations made with reference to the subject under consideration.† There are some other variatians in pitch which are of practical interest. The pitch of vesicular respiration at the upper part of the chest is higher than at the lower. This I have noted in eleven observations, which were all that have been made relative to this point. The pitch does not appear to be sensibly raised by increasing the force of the respiratory movements. It seems to remain the same in ordinary and forced respiration. This I have noted in eight observations, being all that were made relative to

---

* The sound of expiration is oftener heard when the vesicular sounds are exaggerated in supplementary respiration. In the clinical observations of cases of pneumonitis, the presence of a sound of expiration on the healtny side, and the lowness of pitch compared with the sound of inspiration, are noted in several instances. See Appendix First Series. Cases of Pneumonitis.

† The continuousness of the expiration with the inspiration is noted in several of the clinical observations, in which the lungs on one side were free from disease. See Appendix, First Series. Cases of Pneumonitis.

the point The vesicular respiration has a sensibly higher pitch at the summit of the right than of the left chest, in a large proportion of individuals. Of fifteen examinations relative to this point, the disparity just noted was found to be more or less marked in eleven instances, no difference between the two sides being apparent in the remaining four. The practical bearing of the several facts stated in this paragraph on physical exploration in disease will be at once obvious.

In conclusion, while the tracheal and bronchial sounds were found to be essentially the same in character, the circumstances distinguishing each from the other being rather incidental than intrinsic, the vesicular respiration, on the other hand, contrasted with the two former, exhibits some striking points of dissimilarity. It has that inexpressible peculiarity distinguished as the vesicular quality. The inspiratory sound is lower in pitch. The expiratory sound, when heard, save in a limited situation, is lower than tne sound of inspiration, the reverse of this being true of the tracheal and bronchial sounds. Distinctive features less prominent, but important, are the greater length of the inspiratory sound, the shortness of the sound of expiration, the continuity of these two sounds, and the fact that in a large proportion of cases the sound of inspiration alone is appreciable.

*Variations of Pitch in Respiratory Sounds in cases of Disease.*—In treating of morbid variations, it will be most convenient to consider, under separate heads, the different diseases in which they have been studied. The divisions will then correspond with those of the clinical observations given in the Appendix to this memoir.

*Pneumonitis.*—The present inquiry respecting pitch variations of sound has no reference to râles. The crepitant râle in the first stage of pneumonitis frequently drowns other sounds. The modifications which relate to the present subject are incident to the solidification belonging to the second stage of the disease. More or less crepitation, as is well known, may continue into this stage, being heard at the end of the inspiratory act. Under these circumstances, enough of the respiratory sound may be heard, before it is lost in the crepitation, for the pitch to be compared with that of the respiratory sound on the healthy sid.

The observations pertaining to pneumonitis, among those which I have collected, amount to *twelve*. This number might have been increased since my attention has been directed to this subject, but it is probably sufficient for the present object. In each of these observations, the pitch of respiration was notably high, presenting a striking contrast, in this respect, with the respiration on the healthy side. The disparity, as I have noted in one of the observations, is sometimes as obvious as between a fife and German flute. Of these *twelve* observations, an expiratory sound was more or less developed in *eight* instances. In *two*, a sound of expiration was not appreciable; in *one* instance, it was appreciable, but too feeble for its characters to be studied, and in *one* instance the fact as to the presence or absence of the expiratory sound is not noted. The pitch of the sound of expiration was higher than that of inspiration in every instance in which attention was directed to this point, save *one*. In the excepted instance, the observation was made in an early stage of the disease; the crepitant râle persisting, the expiratory sound was feeble, and the fact of its being lower is stated somewhat distrustfully. The expression is, it appears to be lower in pitch. The correctness of this observation seems to me open to doubt, for, as I have had occasion to notice, there is a liability to err in estimating the pitch of a faint sound of respiration. Its feebleness may, without due attention, give rise to the impression that it is lower in pitch. With this doubtful exception, the elevation of the pitch of expiration over that of inspiration was uniform, wherever the expiratory sound was sufficiently appreciable to study its characters.

Aside from what relates to the above pitch modifications, the facts noted with respect to other characters are as follows: The inspiration was shortened in every instance in which attention was directed to this point. This is noted in *five* observations. An interval was observable between the sounds of inspiration and expiration in all the observations containing information on this point: viz., in *six*. The expiration was prolonged whenever attention was directed to this point. It is noted in *five* observations.

The characters, then, belonging to the respiratory sounds incident to pulmonary solidification in pneumonitis, are, elevation of the pitch of both inspiration and expiration; a higher pitch of expiration than of inspiration, whenever the former is heard, almost if not quite uniformly; a shortened inspiratory sound; a prolonged sound of expiration; and an interval between the sounds of inspiration and expiration.

The respiration in the second stage of pneumonitis, as is well known is sometimes characterized as *tubular*, and sometimes as *bronchial.* As contrasted with the vesicular respiration, the first term is certainly significant. The sound may also, with great propriety, be called bronchial, for, on comparing the several points just mentioned with the different elements entering into the normal tracheal and bronchial sounds, more especially the latter, it is perceived that they are essentially identical. The bronchial respiration in pneumonitis is neither more nor less than the bronchial respiration heard in the healthy chest in certain situations. One of the chief points of difference between the normal bronchial and treacheal sounds, it has been seen, is the greater intensity of the latter. The respiratory sounds in pneumonitis vary considerably, in different cases, in degree. The intensity appears to be a contingent, rather than an intrinsic element. In some instances, the development of sound is fully equal to that heard over the trachea, and it may be even louder than is the treachel respiration in some individuals. This comparison of the respiration in pneumonitis with the tracheal and bronchial sounds would naturally enough lead to inquiries respecting the physical philosophy of the identity in their characters. Such inquiries, however, are entirely foreign to my present purpose. To contrast the respiration of pneumonitis with the normal vesicular murmur would be to repeat the points of difference already noticed in connection with the variations incident to health. The disparity is, of course, based on the same circumstances as that between the tracheal and bronchial, and the vesicular sounds.

*Pleuritis.*—The observations relating to pleuritis were made but in *three* cases. So far as these cases go, they show that, when considerable effusion exists, the sound over the compressed lung is high in pitch, presenting, also, more or less of the other characters belonging to the bronchial respiration. After the fluid has been removed nearly, or quite, by absorption, more or less pleuritic adhesions limiting the expansibility of the affected side, the respiratory sound, having resumed the vesicular quality, is relatively feeble when compared with the sound on the other side which is exaggerated, but a disparity in pitch may not be appreciable. If, however, the quantity of effusion has been large, so as to have distended greatly the chest, and led to considerable contraction after the absorption is effected, then, in connection with some permanent dullness on percussion over the affected side, the pitch of respiration may be higher than on the other side, although the vesicular quality and other characters of the vesicular respiration are resumed.

For the exemplification of these statements the reader is referred to the Appendix, Second Series of Observations.

*Gangrenous Excavation, and pneumothorax with Perforation.*—I have had an opportunity of observing, since my attention has been directed to this subject, the respiratory sounds in but one instance of gangrenous excavation, and one of pneumothorax, both occurring in the same case.

Over, or near the site of the gangrenous excavation, which was considerable in size, the sound was noted to be non-vesicular, and low in pitch; as respects the latter, presenting a striking contrast with the sound heard at the summit of the chest, in the same case, where the lung was solidified by compression. Over the latter, the sound was equally non-vesicular, but high in pitch.

The entrance of air into the pleural cavity through a perforation of about the size of a goose-quill occasioned a sound, non-vesicular, and low in pitch.

For a brief account of the case, together with the autopsical appearances, the reader is referred to the Appendix.

*Tuberculosis.*—The physical signs incident to tuberculosis have relation not merely to the presence of the morbid deposit, but to its amount, and the stage of the affection. The study of the disease, with reference to the principles of diagnosis, thus presents itself under somewhat different aspects. During the past two months, I have recorded observations, with respect to variations in the pitch of respiration, in *twenty-five* cases of tuberculosis. I propose to examine the results of these observations under four heads, conforming to the distribution for the cases into four groups in the Appendix to this memoir. Following this arrangement, I shall consider, *first*, the observations in cases in which the tuberculous deposit was supposed to be small in amount; *second*, when the quantity of the deposit was comparatively abundant; *third*, when the disease had proceeded to the stage of excavation; and *fourth*, when the affection appeared to have been arrested.

I. *Observations in Cases of Small Tuberculous Deposit.*—*Eleven* of the cases fall under this subdivision. In each of these cases, the pitch of respiration was found to be higher over the site of the tuberculous deposit, as determined by relative dullness on percussion. In order to test the reality of this variation, and ascertain whether the perceptions might not be influeneed by preconceptions, in several of the cases included in this and the next subdivision, ausculation with reference to the pitch was practiced prior to percussion, or any other examination of the chest. In every instance, the pitch modification indicated the side on which relative dullness on percussion was afterward found to exist. I have frequently caused this experiment to be made by several young gentlemen, medical students, who have accompanied me in my hospital visits, and with uniform success. That is to say, the difference in the pitch of respiration furnished a ready criterion of the existence, or greater abundance of the deposit on one side, before resorting to percussion or other signs.

In some of the observations, the mere fact of elevation of pitch on one side is stated. I was at first satisfied with simply ascertaining this fact, without attending particularly to the expiration, or other points. In several of the cases, however, the observations were more comprehensive.

It is noted that the sound of expiration was appreciable in *six* cases, and inappreciable in *two;* in *three* of the observations, nothing being stated on this point. The expiration is stated to have been prolonged in *four* cases. In *five* cases, the expiration was thought to be either equal to, or higher in pitch than the inspiration: or, to be exact, in *three* of these five cases it was thought to be higher; in the other *two*, it is stated to have been as high, if not higher.

In some cases the vesicular quality was obviously impaired, in connection with the elevation of pitch; but in other cases, disparity in this respect was not obvious. So, with regard to development or intensity of sound, it was sometimes diminished, but not invariably.

To determine the presence of a small amount of tuberculous deposit, in other words, the diagnosis of incipient phthisis, is frequently one of the most difficult problems in practical medicine. Certainly, the employment of physical exploration with reference to this point requires as much accuracy, care and skill as in any of its applications. Several of the auscultatory signs which have more or less importance as indicating the presence of tubercle, are only occasionally present, and the evidence furnished by them is circumstantial, rather than positive. This remark applies to the signs denoting circumscribed bronchitis, pneumonitis, or pleurisy, in proximity to the morbid deposit; the sibilant or mucous râles at the summit of the chest; the crepitant râle, or friction sound, heard in the same situation. Other signs which are doubtless of importance in this connection, must be regarded as somewhat equivocal, such as the jerking respiration, and a prolonged expiration. The most direct and constant of the signs incident to a small tuberculous deposit is the modification known as the *rude*, sometimes called the *harsh* or *rough* respiration. This modification has relation to the present subject, while, to consider the other signs referred to, in this connection, would be irrelevant.

For the novice in the study and practice of physical exploration, the *rude* respiration is generally, of all the signs, the one most difficult to be apprehended. There is probably no sign which causes the teacher or writer more embarrassment in his efforts to explain clearly. Take, for example, the description by Dr. Hughes, author of a very lucid treatise on physical diagnosis. Speaking of the *rude*, compared with the *vesicular* respiration, he says, it "is the *forte* of the same note, but on a loose and jarring string;" and, again, contrastting it with the normal vesicular murmur, he says, "In the one, the same soft breeze passes through a greater number of trees; in the other, the breeze is increased to a moderate gale." Not only is there a singular indefiniteness in the idea conveyed by this language, but the analogies selected for illustration are defective in correctness. The *note* is not the same in *rude*, as in normal respiration, and the comparison to the gale is calculated to give the erroneous impression that the rude respiration is necessarily louder than the normal, which is so far from being true, that it may be in a notable degree less developed ; the intensity being a variable element, having nothing to do with the distinctive character of the sign. The truth is, these terms *rude*, *rough*, and *harsh*, are unfortunate ; they are not only inexpressive, but tend rather to mislead in the apprehension of the modification referred to. The sound is not intrinsically *rude*, or *rough*, or *harsh*. Even a well-marked bronchial respiration, to which the sound under consideration is an approximation, can hardly be said to have the qualities indicated by these terms. The bronchial respiration is not very unlike in character the endocardial sound which is characterized as a *soft* bellows murmur. Whatever appropriateness the designation has, is based chiefly on the fact that, in the rude respiration, the peculiar expansive, breezy attribute of the vesicular respiration, which has beeen referred to as the *vesicular quality*, is more or less impaired.

To form a correct idea of the modification usually termed *rude*, &c., it must be analytically decomposed, and the nature of its elements determined. It is an approximation to the bronchial respiration. It exhibits an incipient development of the character distinguishing the bronchial from the vesicular respiration. One of the most striking of these characters is the change in pitch. The pitch is raised. The vesicular quality is diminished ; hence it approaches to a tubular or blowing respiration. The inspiration may be somewhat shortened, and occasionally a sound of expiration becomes developed and prolonged, constituting an important rythmical variation. By attention to these several points much will be gained in practically recognizing the modification ; but it is not easy to find a satisfactory title to be substituted for the names generally in use. Of the several elements mentioned here, as in the case of the bronchial respiration, it seems to me the pitch modification is the most striking, and the most readily appreciated, while it is probably the most constant.

The expiration deserves to be distinctly noticed. Considerable importance has been attached to a prolonged expiration, since its occurrence was pointed out as a frequent sign of early tuberculization, by James Jackson, the younger, of Boston, Mass. The best practical authorities have recognized this sign as a valuable contribution to the art of physical exploration. It is not, however, a constant modification. Its absence, therefore, does not furnish ground for the conclusion that tubercle does *not* exist. Moreover, it may exist as a normal peculiarity, and consequently alone it is not perfectly reliable. In the few instances among the cases I have collected in which a prolonged expiration was present, and the pitch noted, it was found to be higher, or as high as the sound of inspiration. Now, in health, over the greater part of the chest, if a sound of expiration be appreciable, it is found to be distinctly lower in pitch than the sound of inspiration. May it not be that the elevation of pitch in expiration has a diagnostic value fully as great, or even greater than when the inspiration is thus modified? May it not be that, in some cases in which the inspiration is not sensibly raised in pitch, the expiration may exhibit a change in this respect, and thus the latter furnish a more delicate sign of the presence of the disease? These are interesting, and possibly important questions, which are to be satisfactorily answered only by an accumulation of observations.

It occurs to me here to remark (what would have been more appropriate in connection with pneumonitis,) that the observations of Jackson and others have showed the expiration to become earliest, and in the most striking degree changed, in the development of the bronchial respiration. This fact would lead to the presumption that, in the modification generally known as the rude respiration, the change would be first and most distinctly declared, in the expiration. The expiration in the development of bronchial respiration is said first to exhibit the bronchial character. Although not hitherto recognized as such, I can have little doubt that the change thus noticed consists chiefly in the elevation of pitch. This produces a more striking change in the expiration than in the inspiration, because the change is really greater in amount. The expiration in health is lower in pitch, while in the bronchial it is higher than the inspiration. The degree of modification is, therefore, greater than in the case of the inspiratory sound.

In conclusion, it is highly important to bear in mind that the pitch of respiration at the summit of the right chest is frequently higher than on the left side, and that this may, or may not coexist with a greater development and prolongation of the sound of expiration on the right side. The practical inference is that these modifications, when present on the right side, are, in themselves, less significant than when they occur on the left side, and, in the former situation, are to be less confidently relied upon, especially in the absence of marked rational evidences of tuberculous disease.

II. *Observations in Cases in which the Tuberculous Deposit was abundant.*—The cases arranged under this head are comparatively few in number, amounting to but *five.* In each of these cases, on the side in which the tuberculous deposit was most abundant, as declared by the relative dullness on percussion, and other signs, the respiratory sound was found to be higher in pitch than on the side in which the morbid product was less in amount. In *three* of the cases, the disparity in the pitch of respiration is simply noted in general terms. In the fourth observation it is stated that there existed a prolonged expiration, with an interval between the sounds of inspiration and expiration. The expiratory sound, however, was so feeble that its pitch was not ascertained.

In the fifth observation, an expiratory sound is stated to have been present, higher in pitch than the inspiration, with an interval between the two sounds.

So far as these observations go they lead to the conclusion that the respiratory sound over the site of an abundant tuberculous deposit, when it is appreciable or not obscured by râles, will be found to be elevated in pitch, and representing more or less of the other characters distinguishing the bronchial respiration.

III. *Observations in Cases of Tubercle advanced to the Stage of Excavation.*—The cases coming under this head are of special interest, having reference to the study of the variations in the pitch of respiration occasioned by the presence of a cavity or of cavities within the chest. It has been seen already, in the single case of perforation of the lung and gangrenous cavity, presented in another division, that cavernous respiration, under these circumstances, was low in pitch. This was true in all the cases of tuberculous excavation embraced in this subdivision. The cases studied with reference to the cavernous respiration are *seven* in number. In one of these cases, however, the existence of cavities which had been predicated on physical signs, was disproved by post mortem examination. This case is included in the series for convenience, and serves to illustrate certain points in diagnosis which are to be carefully attended to, in order to avoid an erroneous conclusion. Of the remaining *six* cases, in *two*, autopsical examinations were made, showing the existence of cavities, and their situation with reference to the parts of the chest where the physical signs of excavation had been determined, as noted in the recorded observation before death. The dissection in both instances was made at my request, by Dr. John C. Dalton, Jr., to whom I am indebted for reports of the morbid appearances. The attention of the reader is particularly invited to the cases which include autopsies. (See observations 1 and 3, under same head in Appendix.) In the *four* cases without autopsies, the existence of excavations and their particular situations, are not, of course, demonstrated. The evidence consists of the physical signs, and other facts pertaining to the histories.

So far as the observations go, they show, as already remarked, that the pitch of the cavernous respiration is low, contrasting in this respect, strongly with the high-pitched bronchial respiration. This is not claimed as a newly discovered fact. It is stated by Dr. Walshe, in the last edition of his treatise on diseases of the lungs and heart; and is implied in the language used by Barth and Roger. It is not, however, dwelt upon by either with much emphasis, and, if I am not mistaken, it does not generally receive, with practical auscultators, attention as a diagnostic criterion.*

The pitch of the expiration, when it enters into the cavernous respiration, does not appear to have attracted notice. In the cases I have observed, in which the sound of expiration was appreciable, the pitch was lower than that of inspiration. This forms a striking feature distin-

* For example, in a treatise on disease of the chest, issued during the present year, I find the following statement: "A naturally cavernous respiration exists over the trachea and larynx."

guishing the cavernous from the bronchial respiration. In the latter, as has been seen, the sound of expiration, when appreciable, is higher, or at least equal in pitch to the sound of inspiration.

The cavernous sound is, of course, devoid of what has frequently been referred to as the vesicular quality of normal respiration. This point is generally noted in the observations. It is a point important to be borne in mind in determining the presence of a cavernous respiration; else the vesicular murmur, with its low pitch of inspiration, followed by a still lower expiration, may be thought to denote an excavation. Obs. 7 (see Appendix) illustrates the liability to error incident, in part, to not observing sufficiently this precaution.

The following elements, then, combine to form the cavernous respiration: A *blowing* sound, that is to say, a sound in which the vesicular quality is absent; a low pitch compared with that of the bronchial sound; an expiration, if present, lower in pitch than the inspiration.

Keeping in view these characters, it is probably easy, in most instances in which there are cavities of any considerable size, to determine, with precision, their particular situations, by ascertaining the limits circumscribing the space over which a well-marked cavernous respiration is found to be present. We are assisted in defining the boundaries of these spaces by the fact that excavations are generally surrounded by tuberculous consolidation which will be likely to raise the pitch, and thus exhibit the cavernous sound in stronger contrast. If, however, this were not true in any instance, but, on the contrary, supposing a cavity to be surrounded with lung giving a vesicular sound, we can generally satisfy ourselves of the lowness of pitch (having already satisfied ourselves of the non-vesicular character of the sound in question,) by comparing it with the normal bronchial respiration heard at the sterno-clavicular junction, or with the tracheal respiration. A cavity at the apex of the lung will be more readily discovered by the respiration if it be anteriorly and superficially situated. It is hardly necessary to add that it must be more or less empty, and communicate more or less freely with the bronchial tubes. To ascertain the pitch and other characters, also, the respiration in the surrounding lung must be free from râles.

Tuberculous excavations, as is well known, generally coexist with a greater or less amount of crude tuberculous deposit. In cases, therefore, which present the physical signs of cavities, we have a high-pitched non-vesicular sound, *i. e.*, the bronchial respiration, more or less, over parts of the chest, at its summit, in which the cavernous variations are absent. The observations in the cases of tubercle advanced to excavation furnish illustrations of this fact.

In searching for the sites of cavities, the stethoscope is obviously preferable, since it enables us to circumscribe better the source of sounds. In immediate auscultation, the sounds are received from a wider circuit. The charter of the respiration, as heard by the latter method, will depend on the predominance of excavations or solidification within a certain distance of the part to which the ear is applied. If a large cavity exists, or several cavities, although the intervening tissue be solidified so as to give dullness on percussion, the pitch of respiration may be lower than on the other side in which the tuberculous disease is less advanced. There, the combination of a low, non-vesicular respiration, with dullness on percussion, affords presumptive evidence of the stage of excavation. This is illustrated by Obs. 1. See Appendix.

The importance of attention to the pitch variations in determining the presence of the cavernous respiration is enhanced by the fact that other physical signs of excavations are inconstant and unreliable. A tympanitic resonance on percussion cannot be depended on. It may be absent, although cavities exist, and may be present without excavations. The cracked pot variety is rarely discoverable, and may be due to the expulsion of air from the bronchial tubes or other morbid conditions. Pectoriloquy, which was regarded by Laennec as a pathognomonic sign, is not only frequently absent, but does not possess that distinctive significance attributed to it by the illustrious founder of auscultation. Between pectoriloquy and intense bronchophony there is no intrinsic difference. In other words, pectoriloquy is but a variety of broncophony, and may occur not only in cases with excavations, but when the lung between a bronchial tube and the ear is in a state of solidification from tubercle or effused fibrin. For this statement I have the authority of

Dr. Walshe. But, if it be substantiated by a sufficient number of observations that a cavernous respiration is uniformly low in pitch, with a sound of expiration lower than the inspiration, these traits, taken in connection with its blowing or non-vesicular character, will suffice for its recognition, and consequently, not only for the diagnosis of the stage of excavation, but the localization of the cavity, the estimation of size, &c.

IV. *Arrested Tubercle.*—During the time I have been engaged in making observations with reference to the present subject, two cases have fallen under my notice in which the tuberculous disease appeared to have been arrested; in other words, the morbid product had not continued to accumulate, nor had the primary deposit advanced through the processes of softening and excavation. The cases have not any special importance in connection with the subject of this memoir. I have given them under a distinct head in the Appendix, because they are distinguished from the other cases by the fact of the disease having ceased to make progress.

In this point of view the cases are not without interest. In the first case the disease was declared a year previous, and some cough and expectoration still remain. In the second case, the rational symptoms marking the period of the tuberculous deposit occurred several years ago, and the patient is entirely free from any symptoms of pulmonary disorder. In both instances the disease has left permanent evidence of slight injury to the lung on one side, consisting of disparity of resonance on percussion, and an elevation of the pitch of respiration.

The arrest of tubercular disease is doubtless much more frequent than has been heretofore recognized; and with the views now entertained by the most intelligent practitioners respecting the pathology of tubercle, and the proper ends of treatment, there is reason to hope that cases of this kind will become more numerous. As one of the causes of a lasting disparity between the two sides of the chest in the physical signs developed by percussion and auscultation, this is to be borne in mind in the examination of patients with reference to existing pulmonary affections.

In the practice of auscultation with the view to pitch variations, the same rule of course obtains which is applicable to physical exploration in general, viz: the sounds of the two sides of the chest, in corresponding situations, are to be listened to in succession, and compared with each other, always recollecting to make due allowance for certain natural deviations which are determined by observations made on persons in health, and also, as just remarked, bearing in mind the changes incident to pre-existing disease. That the respiratory sounds may present strongly marked variations in pitch will be readily understood, if it be observed for a moment how easy it is to breathe audibly with the mouth in unison with musical notes, and even in this way to hum a tune. Almost any one who has any fondness* for music is practically familiar with this. Or, as an illustration, let the pitch of sound be observed when different words or letters are whispered after the ingenious method of representing the variations in pitch of the bellows murmur of the heart, suggested by Bouillaud and Hope. The letters *R. S.*, and the syllable *who*, thus exhibit quite different degrees of altitude in pitch. It is easy to distinguish the difference in pitch among endocardial sounds. There is no greater difficulty in appreciating variations in this respect in pulmonary sounds. A musical ear is doubtless an advantage, as also some musical cultivation; but neither is absolutely essential. A medical student, who has been accustomed to follow me in my examinations, and who is unable to distinguish one tune from another, finds no difficulty in recognizing the pitch variations in respiration, and in several instances has made and noted observations by himself which have corresponded with mine. The practice of comparing the pitch of respiratory sounds doubtless leads to an increased facility in directing the attention to, and clearly perceiving variations. This I have found in my short experience, since I have been particularly interested in the subject.

In conclusion, the more important practical deductions submitted in this section are recapitulated in the following summary. These deductions, I would again repeat, are

---

* The pitch of respiratory sound may be readily imitated by modulating, with the lips, breath sounds in the mouth. I have sometimes found this useful in comparing the pitch of sound in the two sides of the chest.

submitted as propositions to be confirmed, enlarged, or corrected. by further investigations:

1. In the second stage of pneumonitis, the inspiratory sound is high in pitch, followed by an expiratory sound, which is frequently, if not generally higher in pitch than the sound of inspiration; these traits being found in conjunction with more or less of the other characters which belong to the bronchial respiration.

2. In cases of small tuberculous deposit, or incipient phthisis, the most striking modification of the respiratory sound is the elevation of pitch. This elevation of pitch is an important element of what is generally known as the *rude*, *rough*, or *harsh* respiration. If an expiratory sound be appreciable under these circumstances, it may be as high, or higher in pitch, than the sound of inspiration, and the variation of pitch in the former is greater, inasmuch as the pitch of expiration in the normal murmur is lower than that of inspiration. Elevation of the pitch of expiration, therefore, may be found to be valuable as a sign of incipient phthisis in some cases in which the variation in inspiration is not marked.

3. If the tuberculous deposit be more abundant, the pitch of respiration is in a more marked degree elevated. The expiratory sound, if appreciable, will be more likely to be as high, or higher in pitch than the sound of inspiration. More or less of the other characters of the bronchial respiration are at the same time present.

4. In pleurisy with effusion, the pitch of respiratory sound is elevated, in conjunction with more or less the characters of the bronchial respiration, over the parts of the chest lying above the compressed lung. In cases of large effusion, after its complete removal by absorption, the affected side may continue to present a variation in pitch, the symmetry of the two sides being permanently impaired, in this respect, after the vesicular quality of respiration is regained.

5. In cases in which tubercle has advanced to the stage of excavation, the site of a cavity of considerable size is indicated by a blowing sound, low in pitch with an expiratory sound (if appreciable) lower in pitch than the sound of inspiration. These traits constitute the elements of the cavernous respiration, and the cavernous respiration is the most constant and reliable of the signs of an excavation.

If the cavity be very large, or there are several cavities, the respiration may be modified to such an extent that, on immediate auscultation, over the whole summit of the chest, it may present the cavernous characters. This may be the case while dullness on percussion shows the existence of more or less solidification in connection with the cavities. The coexistence of relative dullness on percussion, and a low-pitched blowing respiration, denotes the predominance of excavation.

The cavernous respiration may also be present in cases of excavation from circumscribed gangrene, and in pneumothorax with perforation.

6. In arrested phthisis, the traces of the disease may be manifested by a permanent variation in the pitch of respiration, in connection with more or less dullness on percussion at the summit of the chest on either side.

### APPENDIX.—CLINICAL OBSERVATIONS.

The following account of clinical observations embraces a synopsis of the characters of respiratory sounds relating to the subject of the foregoing essay, as noted at the time the examinations in the cases severally were made. Aside from these, details pertaining to the histories of the cases will be introduced, only so far as they seem to be important with reference to the diagnosis, the stage of the disease, or the appearances found after death. The object will be to condense as much as possible, with due regard to the points just mentioned.

For convenience of reference, the observations are distributed into different nosological groups; cases of each disease forming a distinct series. The classification of the observations corresponds with the arrangement of subjects in the foregoing essay, so that reference from either to the other may be easily made.

### FIRST SERIES.—PNEUMONITIS.

*Observation* 1.—Dec. 30, 1851. Hospital patient. Pneumonitis affecting lower lobe of right lung. Second stage of the disease. Loud tubular respiration over the site of the solidified lung. Pitch of respiration notably high.

*Obs.* 2.—January 27, 1852. Wm. Hasmer. Hospital patient. Disease of seven days' standing. Flatness on percussion over the lower third of chest, posteriorly, on the left side. The respiratory sound is bronchial or tubular, the inspiration somewhat shortened; a prolonged expiration is present, with an interval between the sounds of inspiration and expiration. Over the inferior third of the right side, posteriorly, the respiration is vesicular, supplementary, without any sound of expiration. The pitch of sound on the left side, compared with the right, is in a marked degree high, the contrast nearly as striking as between the sound of the fife and the german flute.

*Obs.* 3.—Jan. 29. George Young. Hospital patient. Has been confined to bed four days. Dullness on percussion exists over the inferior third of the left chest. Over this portion the respiratory sound is notably higher in pitch than over the corresponding region of the right side. The inspiration is somewhat shortened. There is a faint but prolonged expiration, and an interval between the two sounds.

*Obs.* 4.—Jan. 30. Mary Gaball. Hospital patient. Disease occurring as complication of continued fever, third day after taking to the bed. Pneumonitis confined to lower lobe of the right lung. Inferior third of right chest, posteriorly, flat on percussion. The pitch of respiration over this region is notably higher than on the corresponding region of the left side. The expiration is somewhat prolonged, and higher in pitch than the inspiration. A short interval between inspiration and expiration.

*Obs.* 5.—Wm. Wrick. Hospital patient. Disease affecting upper and middle lobes of the right lung. Respiration on the right side, posteriorly, bronchial; the pitch high, inspiration shortened, and the expiration prolonged. The pitch of the expiration is notably higher than that of inspiration. Anteriorly, the respiratory sounds on the right side too feeble to determine the pitch, &c.

On the 9th of Feb., the respiratory sound on the right side anteriorly, sufficiently developed to study characters. It is bronchial, higher in pitch than on the left side; expiration prolonged; inspiration shortened, and an interval between the two sounds. The pitch is distinctly higher than on the left side. On the left side the respiration is supplementary. A faint sound of expiration is appreciable, continuous with the sound of inspiration, and apparently lower in pitch than the inspiratory sound.

*Obs.* 6.—Feb. 10. Catherine Finn. Hospital patient. Disease affecting middle lobe of right lung. In first stage. Crepitant rale in mammary and axillary regions. The crepitation obscures the recognition of pitch. On the left side the respiration is supplementary. An expiratory sound is present on this side, continuous with the sound of inspiration, and notably lower in pitch than the inspiratory sound.

Feb. 11. Crepitant rale still heard in mammary region of right side. Posteriorly over the middle third of the right side, there exists moderate relative dulness on percussion. A feeble crepitant rale is heard in this region, but not enough to obscure the pitch of respiration, which is notably higher than over the corresponding region on the left side. The expiratory sound over the middle third of the right chest, posteriorly, is feeble, and appears to be lower in pitch than the sound of inspiration.

*Obs.* 7.—Feb. 12. Michael Ru ssell. Hospital patient. Eighth day of disease. Posteriorly, over middle and lower thirds of right chest, flatness on percussion. In these regions respiration notably higher in pitch than over the corresponding situation on the left side. The sound of respiration is too feeble to determine the pitch.

*Obs.* 8.—Feb. 12. Philip ———. Hospital patient. Disease of a week's standing. Marked dulness on percussion over the lower and right middle thirds posteriorly. Respiratory sound in these regions, in ordinary respiration scarcely appreciable; on forcible respiration more developed, without an appreciable sound of expiration. The inspiration is higher in pitch than on the left side.

On the left side the inspiration is followed by a continuous sound of expiration, which is lower in pitch than the sound of inspiration.

*Obs.* 9.—Feb. 12. James Whalen. Hospital patient. Disease affecting the upper and middle lobes of right lung, of nine days' standing. Flatness on percussion exists over the upper and middle thirds anteriorly, and over the whole side posteriorly. Percussion over the lower third anteriorly, on the right side, elicits clear resonance. Anteriorly, over the upper and middle thirds, a crepitant rale is heard at the end of the inspiratory act.

Over the right side, posteriorly, the respiratory sound is notably high in pitch, and quite intense over the whole side. The sound of expiration is prolonged to an equality in duration, with the inspiration, and there is an interval between the two sounds. The expiratory sound is higher in pitch than the inspiratory. This is the more rmaked the higher up the chest the ear is applied.

*Obs.* 10.—Feb. 13. Jane McNolty. Hospital patient. Disease occurring as a complication of typhus, having taken to bed six days ago. Marked relative dulness on percussion exists over the middle third of left side, posteriorly, and in the axillary region of same side. Over the inferior third, anteriorly and posteriorly, a gastric tympanitic resonance is transmitted. The crepitant rale is heard in the axillary region at the end of the inspiration. Over the middle third, the inspiratory sound is moderately loud, with no appreciable sound of expiration. It is somewhat shorter than the sound of inspiration over the corresponding region of the right side. It has less of the vesicular quality, and is notably higher in pitch. In the latter respect the disparity between the two sides is most marked. A faint crepitant rale is heard at the inferior portion of the right chest posteriorly.

*Obs.* 11.—Feb. 13. John Jarry. Disease of a week's standing. Marked relative dulness on percussion exists over the lower third of right chest posteriorly, and distinct broncophony. No rales observed. The respiratory sounds on both sides quite feeble. Over the lower third of the right side the pitch is notably higher than over the corresponding region of the left side. It is notably higher than over the superior portion of the chest on the same side. The inspiratory sound over the lower third of the right side is relatively shorter in duration. There is a sound of expiration obviously higher in pitch than the sound of inspiration, with an interval between the two sounds. On the left side there is a short, continuous sound of of expiration, which is obviously lower in pitch than the sound of inspiration.

*Remarks.*—In this case, two medical students,* who had been present at previous examinations of patients, and were accustomed to verify the results of my observations, were requested to explore and compare the relative pitch of respiration on the two sides, and the relative pitch of the inspiration and expiration on both sides. Without being acquainted with the result of each other's exploration, or of mine, the conclusions in the three instances were found to be the same. The foregoing statement is noted in connection with the observation.

---

* Mr. J. R. Smith and Mr. Charles Ap. A. Bowen.

*Obs.* 12.—Feb 19. Fitz Morris. Hospital patient. Fourth day of disease. Disease seated in the lower lobes of right lung. Crepitant rale was apparent yesterday, and is now heard at lower part of chest posteriorly. Broncophony over these regions. The respiratory sound is loud, non-vesicular, on high pitch compared with the sound on the left side. An expiratory sound is heard, higher in pitch than the sound of inspiration, with an interval between the two sounds. On the left side, no sound of expiration is appreciable.

SECOND SERIES.—PLEURITIS.

This series will embrace but three cases; in two of which the patients were laboring under the subacute form of the disease, with in one case considerable, and in the other large effusion. In the remaining case, the patient had recovered from chronic pleurisy, and the variations were, therefore, those incident to the permanent changes left by the disease.

*Obs.* 1.—Jan. 4. Conrad Reushling. Hospital patient. Chronic pleuritis affecting the left side. The lower and middle thirds of this side, anteriorly, are flat, and the upper third relatively dull on percussion. Posteriorly, lower third flat, and the upper and middle thirds dull. Over the upper and middle thirds posteriorly, respiration is feeble, and no sound of expiration appreciable. Over the right side the respiration is supplementary. The pitch of sound on the left side, posteriorly, is notably high. This aside from feebleness, is the most striking point of disparity on comparing with the left side. Anteriorly, over the right chest, no sound of respiration appreciable, except just below clavicle, and here the pitch was obscured by dry crackling.

Feb. 1. Up to this date he progressively improved, and had become sufficiently restored to be discharged from the hospital. On the left side, at the summit, there is now slight dulness on percussion; at the inferior portion the dulness is greater. The left chest is one-half an inch less in circumference than the right. The left shoulder is somewhat depressed. The left side expands less at the summit than the right. The respiratory sound on the right side, is exaggerated, supplementary. On the left it is relatively feeble. In both sides, the respiratory sound has the vesicular quality, a feeble sound of expiration being appreciable, and the duration of the respiratory sounds being apparently equal. After careful examination, I cannot satisfy myself of a disparity of pitch between the two sides.

*Remarks.*—The following remarks were appended at the time of the last observation. This case affords an opportunity of comparing an exaggerated, with a relatively diminished vesicular sound. The physical and rational signs show that the patient has labored under subacute pleurisy, from which he has nearly recovered. There is slight condensation of the left lung from compression, and less expansibility from pleuritic adhesions. Here are two opposing circumstances as respects their probable influence on the respiration. The defective expansibility might be expected to occasion a pitch of sound somewhat lower than that on the right side, the respiration in the latter being, at the same time, exaggerated. On the other hand, the condensation would be expected to elevate the pitch. These apparently conflicting circumstances serve to antagonize each other, the fact being that the respiratory sound at the summit of the chest on both sides is not far from equal. It may be inferred from this case that exaggeration of the vesicular murmur does not tend, in a marked degree, to elevate, nor, on the other hand, a feeble vesicular murmur to lower, the pitch of sound.

*Obs.* 2.—Feb. 8. Ortmann. Hospital patient. The effusion, in this case, was large, compressing the lung into a small space at the upper and posterior part of the chest. The disease was in the right side. No respiratory sound is to be heard save in the clavicular, post-clavicular, supra-spinous, and interscapular regions. Over the supra-spinous, and interscapular regions, the sound is feeble, non-vesicular; the expiration louder and higher in pitch than the inspiration. The inspiration is shortened, the expiration prolonged, with an interval between the two sounds.

On the left side, in the corresponding regions, the respiratory sound is supplementary. A sound of expiration is heard, which is about one-fourth the length of the inspiration. The sound of expiration follows that of the inspiration without any appreciable interval. In pitch, the expiration on this side is distictly lower than the inspiration.

In pitch, the inspiration, as well as expiration, is higher on the right than on the left side; but this is more especially marked in the expiration.

*Obs.* 3.—Feb. 10. Eliza Moore. This patient, eighteen months ago, had subacute pleurisy of the left side. The chest, on this side, was greatly distended, the heart being dislocated, so that its pulsations were seen and felt to the right of the sternum. She has remained in hospital up to this present time, but is now quite well, and has been employed as a domestic, performing hard labor, such as washing, scrubbing floors, &c. The left chest is considerably contracted. The impulse of the heart is now felt somewhat above the normal situation.

The left chest is relatively dull on percussion. The respiratory sound has the vesicular quality, but is higher in pitch than on the right side. There is a short, feeble, continuous sound of expiration. The pitch of the expiration appears to be lower than that of the inspiration. Near the right sterno-clavicular junction, the respiration is bronchial; the inspiration high in pitch, with an expiration higher than the inspiration. It is more intense, and on a higher pitch on the right than on the left side, near the sterno-clavicular junction; this being the reverse of the vesicular respiration, which is higher in pitch on the left side.

The pitch of the vesicular respiration is not increased by a deeper and stronger act of respiration.

THIRD SERIES.—GANGRENE AND PNEUMOTHORAX.

Under this head, a single case only will be embraced. This case occurred in private practice. The patient, aged about forty-five, had suffered, for between one and two years, with occasional attacks of epilepsy. He had for many years been addicted to the use of stimulants. The mental faculties were somewhat impaired, the memory especially. On the 28th of January, after a severe epileptic paroxysm, pulmonary symptoms became developed. The physical signs of pneumonitis and pleurisy being absent the pulmonary affection was supposed to be bronchitis. On the 5th February, when I again visited in consultation, there were the physical evidences of solidification of lung at the middle third of the right chest anteriorly and posteriorly, and a dirty expectoration with a characteristic fetid odor. It was evident that the patient was laboring under pulmonary gangrene. On the 8th, I again saw the case, and at this time the fetid expectoration had ceased, and he presented the physical signs of pneumothorax, viz: tympanitic resonance at the upper and middle thirds of the chest anteriorly, and metallic tinkling at the inferior third, just below the nipple. On the 9th I noted as follows: "The physical exploration was brief, owing to the great prostration of the patient. On the right side, over the upper and middle thirds, anteriorly, and on the upper third, posteriorly, percussion gives a tympantic resonance. Below, flatness on percussion. The respiration over mammary region is non-vesicular, and low in pitch. Above this region, in front, the sound is indistinct. On the upper third, posteriorly, the respiration is high in pitch. Below, feeble, but low in pitch."

The patient died on the 10th, and the chest was examined on the 13th, by Dr. John C. Dalton, Jr., who has furnished the following statement of morbid appearances:—

"Autopsy of Mr. S——, February 13th, 1852. On opening the cavity of the right pleura, a considerable quantity of rather fetid gas escaped, and on raising the sternum the same cavity was found to contain about two pints of a dingy, yellowish-gray purulent fluid. The pleural surface at the anterior part of the chest was covered by a thin coating of recent lymph.

"The right lung reduced to about one-fifth of its natural size, was compressed against the spine and the posterior wall of the chest. Before removing the fluid from the pleural cavity a pipe was inserted into the trachea, and on inflating the lungs the air escaped freely in large bubbles, from a point situated toward the posterior part of the right lung, about the junction of its upper and middle thirds. The right lung, removed from the chest, was found to be completely carnified by pressure, except in those parts o^cupied by disease. No air cells were visible anywhere, and the whole lung was destitute of crepitation. The lobes were adherent to each other by recent lymph. The upper third of the lower lobe was occupied by gangrene, which had reached an advanced stage, the pulmonary tissue forming a soft, shreddy, disintegrated mass, of a fetid odor, and a dirty, grayish color and infiltrated with purulent fluid. A gangrenous cavity of considerable size, had apparently existed at this spot before the compression of the lung. The opening by which it had communicated with the pleural cavity was about the size of a goose-quill, and situated posteriorly at the very uttermost portion of the lower lobe. The limits of the gangrenous portion of the lung, were well defined, but only a very little inflammation existed in its neighborhood, the solidification of the pulmonary tissue, except just outside the limits of this gangrenous cavity, being entirely of a passive character, and due to its compression by the pleuritic effusion. The left lung and pleura were healthy. There was no tubercle anywhere. Signed J. C. D.

*Remarks.*—On comparing the above appearances with the physical forms noted before death, it is evident that the non-vesicular respiration, with a high pitch, which was heard at the summit of the chest posteriorly, was due to the consolidated lung in this situation. The non-vesicular sound, low in pitch, heard in the mammary region, is attributable to the entrance of air into the pleural cavity through the perforation. The low pitched non-vesicular sound heard at the inferior posterior part of the chest was probably owing to the entrance of air into the gangrenous cavity.

### FOURTH SERIES.—TUBERCULOSIS.

For convenience of reference, the cases of tuberculosis will be distributed into four subdivisions, according to the amount and stage of the disease, as follows:

1. Small tuberculous deposit.
2. Abundant tuberculous deposit.
3. Tubercle advanced to excavation.
4. Tubercle arrested.

### 1. *Small Tuberculous Deposit.*

*Obs.* 1.—January 4. George Laver. Hospital patient. Slight dulness at summit of right chest. Respiration at summit of right chest relatively feeble, without any aberration of rhythm. The elevation of pitch compared with the left side, is distinct.

*Remarks.*—The symptoms in the history of this case pointing to the existence of tubercle are as follows:—cough for one and a half years, with slight expectoration. Slight hæmoptysis. Respirations increased in frequency ranging from twenty-four to thirty-six. The patient was not much emaciated nor greatly debilitated; had not been subject to night perspirations or pleuritic pains. Countenance not notably pallid; no febrile movement.

This was one of the earliest observations relative to the subject, when I was satisfied simply to ascertain the pitch of the respiratory sound over the site of the tubercle as determined by percussion, without directing attention to comparative pitch of expiration, and other points. This remark will account for the limited scope of the observations in several other instances.

*Obs.* 2. —Jan. 4. Slayter. Hospital patient. At the summit of right chest marked relative dulness on percussion. The pitch of respiration is higher than on the left side. Aside from this, a disparity is not apparent, except in the relative feebleness of sound on the right side. No murmur of respiration is appreciable.

Jan. 27th. The following was noted as a distinct observation, without reference to the former: I find relative dulness at the summit of the right chest anteriorly. Over this situation the respiratory sound is relatively feeble. I cannot discover a difference of inspiration between the two sides, nor is there a sound of expiration appreciable on either side. Aside from relative feebleness, the elevation of pitch appears alone to distinguish the respiration on the right from that on the left side.

Feb. 9. The difference in the vesicular quality between the two sides is not marked. The marked difference is in pitch.

*Remarks.*—The symptoms, in the history of the case, pointing to tubercle, are as follows: cough of four months' continuance, with small expectoration. Night perspirations. Is debilitated and pallid, but not greatly emaciated. Pulse 88. Respiration 18. Has not had hæmoptysis, nor complained of pleuritic pains.

*Obs.* 3.—January 28th. Aaron Young. Hospital patient. Slight relative dulness on percussion in right infra-clavicular region. The respiration in the right infra-clavicular region is higher in pitch than in the corresponding region of the left side. The expiration is prolonged, with an interval between the inspiration and expiration. In the left infra-clavicular region the respiration is lower in pitch. There is a short sound of expiration, with a short interval between the two sounds. The inspiration is longer than on the right side. Slight broncophony exists in the right infra-clavicular region.

March 2d. At summit of right chest, the expiration as long as the inspiration. Inspiration and expiration about the same in pitch. Near the sterno-clavicular junction, on both sides, loud bronchial respiration, but much more developed, and higher in pitch on the right side. Expiration more developed on this side, the pitch about the same as that of the inspiration.

March 3d. Expiration and inspiration at summit of right chest apparently about similar in pitch, the expiration notably prolonged. At the summit of the left chest a sound of expiration is appreciable, notably lower in pitch than the inspiration. The contrast between the two sides consists in an elevation in the pitch of both sounds on the right side ; a prolonged expiration, about equal in pitch to the inspiration on the right side, while the pitch of the expiration is lower than that of the inspiration on the left side.

*Remarks.*—I give this among the cases of tuberculosis, but I do not feel positive with respect to the diagnosis.* On another examination made on the day I am writing, viz : March 8th, I find the same physical signs, with marked relative dulness over the right scapula below the spinous ridge. The symptoms in the history which relate to phthisis are cough, since January, with moderate expectoration. No hæmoptysis or pleuritic pains. He entered the hospital for cough and general debility, Janu-

* The correctness of the diagnosis was subsequently demonstrated.

ary 13th. Afterward he contracted typhus, from which he is now convalescent. His cough and expectoration are less at the present time than when he entered. The physical signs at the summit of the right chest may possibly be due to a normal disparity, which, as has been seen, frequently exists to a greater or less extent—in this instance, perhaps unusually marked; or they may be due to uniform enlargement of the bronchi on the right side.

*Obs.* 4.—Jan 30. Felata Kyser. Hospital patient. Slight relative dulness of resonance in left infra-clavicular region. The dulness is more marked over the left scapula, and in the latter situation there is a distinct broncophony. The pitch of respiration is in a marked degree higher on the left than on the right side, at the summit of the chest anteriorly and posteriorly. The expiration is not prolonged. The elevation of pitch is the chief source of disparity between the two sides.

*Remarks.*—The symptoms pointing to tuberculous disease are cough, with small expectoration, pleuritic pains; occasional night perspirations; respiration 28; the pulse is 70. She had not had hæmoptysis.

*Obs* 5.—Feb. 1. Harmon Kramp. Hospital patient. Relative dulness of resonance, and distinct broncophony, in the right infra-clavicular region. The pitch of respiration, in this situation, is notably higher than on the left side. The inspiration is somewhat shortened, the expiration prolonged, and higher in pitch than the inspiration, with an interval between the two sounds.

*Obs.* 6.—Feb. 2. Mrs. R. Out-patient. On auscultation, before percussing, the respiration is found to be notably higher in pitch in the right, than in the left infra-clavicular region. Aside from this disparity in pitch, the inspiration on the right side is somewhat shortened, with a short sound of expiration; on the left side no sound of expiration is appreciable.

Slight relative dulness of resonance exists in the right infra-clavicular region, and distinct broncophony.

*Remarks.*—The symptoms in the history of this case pointing to tuberculosis are, cough, troublesome at night, almost entirely without expectoration; pleuritic pains; considerable emaciation; pallid complexion; night perspirations; tuberculous fever. Respiration 28.

*Obs.* 7.—Feb. 12. Patient at almshouse; name not noted. Slight dulness on percussion at the summit of chest on the left side. Below, resonance equal on both sides. The respiratory sounds, at the summit of the chest, on both sides, are about equal in intensity. There is an equally short, faint, scarcely appreciable expiration on both sides. The pitch, however, on the left side is notably higher. The vesicular quality is also somewhat diminished on the left side.

*Remarks.*—The symptoms in this case were not noted. It is simply stated that the history and symptoms point to tuberculosis.

*Obs.* 8.—Feb. 16. Mrs. R. Private patient. Marked relative dulness on percussion over the right scapula, with broncophony. Slight relative dulness in right infra-clavicular region. The respiratory sound at the summit of the chest is notably higher in pitch than on the left side. There is a faint sound of expiration, which is prolonged, and appears to be higher in pitch than the inspiration. On the left side, the sound is lower in pitch, the vesicular quality is more marked, and no sound of expiration is appreciable.

*Remarks.*—In this case, I was led to the site of dulness on percussion, by the signs developed by auscultation, resorting to the latter method before the former. There are few symptoms denoting, directly, pulmonary disease in the case. The patient has been recently confined, having had three children in the space of five years; lactation protracted during the last pregnancy up to the period of quickening. Since her confinement she has had chills, with irregular paroxysms of febrile movement. She is considerably emaciated and prostrated, presenting a morbid aspect. Cough has been present, only occasionally, and is not a prominent symptom. The expectoration is insignificant; she has not had hæmoptysis, nor distinct pleuritic pains; the secretion of milk was tolerably abundant, but weaning was speedily resorted to, and she has since improved in strength and appearanc, without any further development of pulmonary symptoms. These remarks were written three weeks after the above observations were noted.

*Obs.* 9.—Feb. 22. Murdoch Gillis. Hospital patient. On immediate auscultation, the pitch of respiration is higher on the right side at the summit. This is more marked on mediate auscultation. The pitch in the right infra-clavicular region is highest toward the acromial angle. There is here a prolonged expiration higher in pitch than the inspiration.

*Remarks.*—The signs on percussion are inadvertantly not embraced in the observation. It is noted that Mr. Smith, one of the resident students at the hospital, had made and recorded the results of his examination prior to mine, which were afterward compared with the above and found to correspond.

The symptoms in the history pointing to tubercle, are as follows: cough of eleven months' standing; moderate expectoration; considerable emaciation, pallor and debility, and, of late, slight perspirations at night. Has not had hæmoptysis nor pleuritic pains. Pulse 108; respiration 30.

*Obs.* 10.—Feb. 24. Robert Evans. Out-patient. Slight dulness on percussion in right infra-clavicular region. The pitch of respiration in this region is higher than on the left side. There is no appreciable sound of expiration in this situation.

Posteriorly, over the scapula above and below the spinous ridge, there is marked relative dulness on percussion. The respiratory sound in these regions is very feeble; but, in forced respiration, the pitch is determined to be notably higher than on the left side, and a sound of expiration is heard as high or higher than the sound of inspiration. Moderate but distinct broncophony exists at the summit of the right chest, anteriorly and posteriorly, and not on the left side.

*Remarks.*—The patient has had a cough for nine months, with small expectoration. Has lost considerable flesh and strength, and is incapacitated for labor, but is able to be up, and to walk out of doors. Respiration 24. Other details pertaining to the history were not recorded.

*Obs.* 11.—March 3. Louisa Stately, aged 8 years. Hospital patient. Distinct dulness on percussion in left infra-clavicular and over the scapular regions. The respiratory sound is more developed in the left than in the right infra-clavicular region. A difference in duration of the inspiration, or in the vesicular quality is not appreciable. There is a feeble sound of expiration on both sides. The chief point of disparity pertains to the pitch, and this is marked. It is higher on the left side. The expiration on this side appears to be about equal in pitch to the inspiration, while it is lower than the inspiration on the right side. The pitch is also notably higher on the left than on the right side, over the scapular region.

*Remarks.*—This patient has had cough for several months, and, once, slight hæmoptysis. She is thin and pallid. Before my examination in this case, Mr. Bowen, medical student, had made an examination with reference to the pitch of sounds, and recorded the results, which were found afterward to correspond with those obtained by me, save so far as the latter relate to the expiration, of which he made no note.

2. *Abundant Tuberculous Deposit.*

*Obs.* 1.—Jan. 4. Biselay. Hospital patient. Marked relative dulness of resonance at the summit of the left chest posteriorly, and of the right chest anteriorly. Respiration, on both sides, tubular, with prolonged expiration.

Distinct relative elevation of pitch at the summit of the left chest posteriorly, and of the right chest anteriorly.

*Remarks.*—In the above, as in other observations made shortly after my attention had begun to be directed to the study of the pitch of respiration, the fact of the disparity of pitch alone was noted, without reference to the expiration and other points.

*Obs.* 2.—Jan. 4. Julia Moniva. Hospital patient. At summit of right chest anteriorly, marked relative dulness on percussion. On auscultation, sonorous râles occasionally on both sides, at the summit of the chest. Respiratory sound more developed on the right side. No expiratory sound on either side. On the right side, distinct elevation of pitch. Distinct broncophony on the right side.

On another examination after the foregoing was noted, the patient in the mean time having coughed and expectorated, I find the respiratory sound more developed at the summit of the left chest, and on comparing the pitch, the elevation is more strongly marked on the right side than before. The disparity in pitch is the most strikiug point of difference between the two sides in this case.

*Obs.* 3.—Jan. 5. Welch. Hospital patient. Marked relative dulness on percussion at the summit of the left chest. Respiratory sound feeble, and tubular on the left side, presenting a marked elevation of pitch compared with the respiration on the right side.

*Obs.* 4.—Feb. 19. Daniel Cuit. Hospital patient. On immediate auscultation, prior to examination by percussion or inspection, the respiration at the summit of the right chest is found to be notably higher in pitch in comparison with that at the summit of the left chest. The inspiration on the right side is shorter than on the left. On the right side there is a feeble, prolonged sound of expiration, with an interval between the two sounds. The expiratory sound is too feeble to determine the pitch. On the left side there is a feeble, short sound of expiration, which is continuous with the sound of inspiration.

Afterward, it was found that the summit of the right chest is notably dull on percussion, and marked broncophony exists on this side.

*Obs.* 5.—Feb. 20. Evelina Potter. Hospital patient. On examination by auscultation, before practicing percussion or inspection, with a view to determine on which side tuberculous deposit might be present, or more abundant, I found, at the summit of the right chest, the inspiratory sound higher in pitch than on the left side; the vesicular quality less; a sound of expiration higher in pitch than the sound of inspiration, prolonged to the same length as the inspiration, and an interval between the two sounds.

At the summit of the left chest the sound of inspiration is feeble, but lower in pitch than on the right side. A prolonged expiration exists on this side, high in pitch, with an interval between the two sounds.

On percussion there is marked relative dulness on the right side over the infra-clavicular and scapular regions.

An occasional sibilant rale is heard in the right infra-clavicular region.

Broncophony exists on both sides, but is much more marked on the right side.

### 3. *Tubercle Advanced to Excavation.*

*Obs.* 1. *With Autopsy.*—Jan. 25. Hugh McMullin. Hospital patient. Marked relative dulness on percussion, and broncophony at the summit of the right chest. Respiration over right chest at summit presents a notable elevation of pitch on comparison with the summit of the left chest. The expiration on the right side is prolonged, exceeding in duration the sound of inspiration. The inspiratory sound is shorter than on the left side and there is a longer interval between the two sounds, but less in duration than on the right side. On the left side the sound of expiration is higher in pitch than that of inspiration.

Jan. 31. Over a space in the right infra-clavicular region, about midway between the acromial and sternal sides of this region, the respiratory sound is lower in pitch than in every direction surronnding this space. A feeble sound of expiration is heard over this space. No pectoriloquy. Above this space, directly upon the clavicle, the respiration is intensely tubular, with prolonged expiration, the pitch quite high, resembling tracheal respiration.

In the left infra-clavicular region, over the space corresponding to that just referred to, (*i. e.* just below the clavicle, about midway between the two extremities,) the respiratory sound has a high pitch, the expiration being prolonged.

The right side presents, on percussion, a higher pitch of resonance, but a hollow, or tympanitic sound.

On immediate auscultation over the summit of the chest the right side gives the lower pitch. It is to be observed in this case, that the side on which the percussion pitch is highest, has the lower pitch of respiration.

Feb. 1. A *bruit de pot fêlé* is to-day distinctly apparent over the space in the right infra-clavicular region, characterized by the gravity of the pitch of respiration. The latter fact is also again verified.

Feb. 18. Confirmed again the statements made in the foregoing observation, and observed, in addition, that over the space in the right infra clavicular region presenting a low pitched sound of respiration, the expiration is lower in pitch than the inspiration. On examination of the left infra-clavicular region yesterday, and to-day, I find a space about midway between the acromial and sternal extremities, and about half an inch below the clavicle, in which the sound is blowing, *i. e.* non-vesicular, and lower in pitch than in every direction surrounding it. There is a sound of expiration here which is lower in pitch than the sound of inspiration.

This case proving fatal on the 21st of Feb., the lungs were examined on the 23d, by Dr. John C. Dalton, Jr., who has furnished the following statement of morbid appearances :—

"*Right lung.*—The upper, and one-half of the middle lobe are entirely without crepitation, being completely solidified except at situation of cavities, by deposits of yellowish, cheesy tubercle, and gray infiltration. The greater part of the upper lobe is occupied by a superficial irregular cavity nearly empty, twice or more as large as a hen's egg, situated quite at the apex, and rather toward the outer part of the lobe. No other cavity of any considerable size in the right lung. The lower lobe crepitates pretty freely, but contains also many small yellowish tubercles. Many miliary tubercles sprinkled over the pleural surface of the lower lobe.

"*Left lung.*—Apex of upper lobe crepitates freely, and is nearly healthy, but its middle portion is extensively solidified by yellowish tubercle, and by well-defined spots of light-red hepatization. About the middle of the anterior portion there is a superficial cavity about the size of a walnut, containing a drachm or so of pure pus. Remainder of the left lung somewhat tuberculous, but less so than other parts."

*Remarks.*—It is evident, on comparing the foregoing morbid appearances with the preceding observations, that the low pitched respiration (consisting of a low inspiration and a lower expiration,) heard over a circumscribed space in either infra-clavicular region, was due to the cavities existing in the lungs on both sides, while the high pitched respiration, heard in the neighborhood of these spaces in every direction, was owing to the solidification of the lung surrounding the excavations. The latter, in other words, was the bronchial, and the former the cavernous respiration. It may seem, at the first glance, that the results of the first of the several examinations conflict somewhat with those of the subsequent examinations. The apparent inconsistency may be readily explained. Auscultation with the stethoscope in certain portions of the right infra-clavicular region, that is, over the portions of lung solidified, would, of course, give a high pitch of sound. That the low pitched respiration, denoting excavations, was not discovered at the first exploration, may have been because the whole of the infra-clavicular region was not carefully examined, or the cavities may have been filled with fluid secretions, and therefore the conditions for the cavernous respiration were not present.

*Obs.* 2.—Jan. 26. James Bell. Hospital patient. On the left side, marked relative dulness on percussion at the summit.

Over the right side, at the summit, the respiration is evidently supplementary, the murmur being intense, but vesicular, without any sound of expiration. The resonance on percussion on this side is quite clear. The pitch of respiration, however, is rather high.

Over the left chest, at the summit, the respiratory sound is lower in pitch than over the right side. The pitch, however, is not uniform over the whole of the summit of the left side. Toward the acromial angle of the infra-clavicular region, over a space of about the dimensions of the thoracic extremity of the stethocope, the pitch is low. At a short distance, in a direction toward the sternum, the pitch is notably higher. Over the latter space, compared with the former, the sound on percussion is in a marked degree dull. The resonance over the former space seems to me somewhat tympantic in character, and the tone is communicated through the stethoscope with a resonance approaching to pectoriloquy.

On applying the ear immediately over the summit of the left chest, the pitch is lower than on the right side.

The history of this case shows copious muco-purulent expectoration.

*Obs.* 3. *With Autopsy.*—Feb. 2. O'Donnell. Hospital patient. On the right side the resonance is relatively dull in a marked degree. On immediate auscultation, the pitch of respiration is found to be notably elevated, with a prolonged expiration, the latter being quite high in pitch. About an inch and a half, on the right side, below the clavicle, toward the acromial angle the pitch is low, devoid of vesicular quality. At the same distance below the clavicle, toward the sternal extremity the pitch is high.

Feb. 13. On examination of this patient to-day, I observed at the summit of the right chest, during the first part of the act of respiration, a tubular respiration loud and high in pitch; and during the latter part of the inspiration, the inspiration abruptly became low, the blowing character being still preserved. The contrast between the two portions of the inspiration was striking. There is a feeble sound of expiration which is low in pitch.

This case terminated fatally a few days after, and the following statement of the morbid appearances of the lung is furnished by Dr. John C. Dalton, Jr.:—

"The right lung is closely confined by old and very firm adhesions throughout its upper portion. Whole of upper and most of middle lobe destitute of crepitation, and solidified by gray (tubercular) infiltration. Lower lobe full of air, but shows also many small, well-defined cheesy and softened yellowish tubercles. Upper lobe toward median line, solid, or with only one or two small cavities. Toward sternal portion there is a large superfical cavity, with irregular walls, capable of holding an egg or more, containing a few drachms of pure pus, and lined throughout by a grayish, semi-transparent false membrane, about a line and a half in thickness; easily scraped off by the edge of the scalpel. This cavity communicates with other smaller ones at posterior part of upper lobe, and also with a branch of the right bronchus about the size of a goose-quill.

"The left lung shows many small, yellowish, cheesy, and softened tubercles,and one cavity at its posterior part nearly or quite empty, and exhibiting the appearance of having been for some time inactive. But very little gray infiltration in the left lung, and it is altogether much less diseased than the right. No effusion in either pleural cavity. (Signed) J. C. D."

*Remarks.*—The low pitched sound of respiration, with the expiratory sound lower than the inspiration heard over a circumscribed space in the right infra-clavicular region, was doubtless due to the cavity discovered in the lung on this side at the autopsy. The high pitched sound in a direction from the cavity toward the sternum was produced by the solidified lung. The latter sound, in other words, was bronchial, and the former cavernous. The combination of the bronchial and cavernous respiration in the act of inspiration is an interesting point in this case, the former due to the current of air in the bronchial tube passing through solidfied lung to communicate with the excavation, and the latter produced by the entrance of air within the cavity.

The examinations were limited to the anterior part of the chest, owing to the great feebleness of the patient A careful exploration over the posterior summit of the chest, on the left side, might have led to the pitch variations incident to the cavity in the posterior part of the left lung.

*Obs.* 4—Feb. 22. John Jervis. Hospital patient. On immediate auscultation, the pitch of respiration is found to be higher in the right infra-clavicular region. The respiration is short and non-vesicular. On immediate auscultation, over a space near the sterno-clavicular junction, the pitch is low, and the sound non vesicular. A sound of expiration is not sufficiently appreciable to determine the pitch. The sound over the space just referred to contrasts strongly with the high pitched bronchial respiration over the sterno-clavicular junction. The chest is depressed on the right side in the infra and post-clavicular regions. It expands less in these situations, on inspiration, than on the left side.

On percussion, the right infra-clavicular region is found to be relatively dull. Over the space referred to, the sound is lower in pitch than over surrounding portions, and somewhat tympanitic. Over this space there is forcible vocal resonance, but not marked pectoriloquy.

Over the right mammary region there exists marked relative dulness on percussion, almost amounting to flatness. There is not much resonance, but comparatively a greater degree on the left side. The respiration on both sides over this region is feeble, but on the right side notably higher in pitch.

*Remarks.*—The disease in this case had existed for about six months. The respirations were 32, and the pulse 128. The comparative lowness of the pitch of respiration, with the absence of the vesicular quality, over a space in the right infra-clavicular region, is presumed to denote the existence of a cavity in that situation. In other words, the respiration in this situation is cavernous.

In this case, at my request, Mr. Bowen, medical student, examined and recorded the results, which were found to correspond with mine.

*Obs.* 5.—March 5. Carlos Aberd. Hospital patient. This patient entered in the last stage of the disease, and was so greatly prostrated that the physical examination was very cursory. Death occurred the second or third day after his admission, and an autopsy was not practicable. It is simply noted, that on the right side, at the summit of the chest, before and behind the respiration is non-vesicular, low in pitch, with an expiration lower in pitch than the inspiration.

*Obs.* 6.—Feb. 26. James Kelly. Hospital patient. The examination for pitch is difficult on the left side, owing to the presence of râles. There exists marked relative dulness on percussion over the left side, at the summit of the chest anteriorly and posteriorly. On the right side, posteriorly, the respiration over the scapula is high in pitch, with a prolonged expiration higher in pitch than the inspiration.

On the left side (the side in which relative dulness exists) over the scapula the sound of respiration is lower in pitch, and devoid of the vesicular quality. An expiration is present lower in pitch than the inspiration.

In the right infra-clavicular region toward the sternal boundary, the pitch is high, and the sound loud, without an appreciable expiration. Toward the acromial border, in the same region, the pitch is low, with an expiration lower than the inspiration. The sound here, however, has the vesicular quality. Percussion over the half of this region in the direction of the sternum is dull, while over the other half it is clear.

In the left infra-clavicular region, râles obscure the pitch save over a space near the sternum. Here the pitch is low, with an expiration lower than the inspiration.

*Obs.* 7, *with Autopsy.*—Jan 27. This case is included among the cases of tuberculosis advanced to the stage of excavation. It was not, however, really a case of that description. The existence of a cavity was predicated upon certain pitch characters, and other signs, but the autopsy proved the diagnosis to be incorrect. Candor requires the observation to be given in connection with the post-mortem appearances. The case is interesting and instructive, showing circumstances which were perhaps well culculated to lead to error of diagnosis. It also illustrates, incidentally, certain points connected with the present subject.

James Caytor, aged 5 years. Hospital patient. On percussion, the resonance in the left infra clavicular region appears to be somewhat tympanitic, but the pitch of resonance is distinctly higher than on the right side. There is a distinct *bruit de pot félé* in the left infra-clavicular region. The pitch of respiration is distinctly lower in the left infra-clavicular region than in the right. There is a feeble expiratory sound on this side, not uniformly, but occasionally appreciable. The pitch is lower in the above situation than on the same side over the mammary region.

Over the right infra-clavicular region, the pitch of respiration is higher than over the left mammary region. Pectoriloquy is not present. In the left infra clavicular region the second rib yields with very little resistance to pressure, in this respect contrasting strongly with the resistance over the corresponding region on the right side.

Jan. 29. Confirmed the foregoing facts. The *bruit de pot félé* is distinct.

The case terminated fatally early in the present month (March,) and the following statement of the morbid appearances of the lung is furnished by Dr. John C Dalton, Jr.:

"The pleuræ of both lungs were rather plentifully sprinkled with tubercular granulations—no effusion or adhesion. There were also a few small tuberculous masses in substance of middle and lower lobe of right lung, yellowish and firm in consistence; no cavities and no softened tubercles. The largest collection of tubercle was just behind the root of the left bronchus, firm and cheesy—about the size of a small English walnut. Bronchial glands slightly tuberculous. Bronchi of natural relative size and length. There was considerable redness of mucous membrane of the left bronchus, with three or four shallow white ulcerations. No tuberculous deposit perceptible at base or in neighborhood of these ulcerations. No similar affection of right bronchus. Apices of lungs nearly or quite free from tubercle. No pneumonia anywhere. (Signed) J. C. D."

*Remarks.*—A cavity was sought for in the left infraclavicular region, supposed to be due to excavated tubercle or pouch dilatation of the bronchus in this situation. This expectation was based on the physical signs above mentioned, viz: the low pitch of respiration, the tympanitic resonance on percussion, and the *bruit de pot fêlé.* The diagnosis was formed with considerable hesitation, because the rational system did not denote extensive tuberculosis advanced to the stage of excavation. The respirations were not accelerated nor labored. There did not appear to be much expectoration. The age of the patient militated against the supposition. The patient was reduced to marasmus, and died by asthenia, evidently from defective assimilation. In spite of these circumstances, the pitch characters, and the cracked-pot, tympanitic resonance, were thought to denote the existence of a cavity.

The source of the error is intelligible. The thoracic walls were very thin, and the respiratory sound was largely developed. The respiration on the right side at the summit, was higher in pitch from the predominance of the bronchial element as a natural disparity. I was not, at that time, so well prepared to attribute a disparity so strongly marked to a normal difference, as since the examination of a number of healthy chests with reference to this among other points. The relative lowness of the pitch on the left side did not arise from a depression on this side, but because the normal elevation of pitch on the right side was in this instance strikingly marked. Instead of a cavity, there existed in the right lung a solid tuberculous deposit of about the size of an English walnut. This was situated directly behind the left bronchus. In this situation it would not evidently be expected to raise the pitch of the bronchial respiration in front on this side, and by making some pressure on the tube it would be likely to render the sound less developed.

How is the *bruit de pot félé* to be accounted for? It is well known that this modification of tympanitic resonance does not universally require a cavity for its production. It has been observed in the second stage of pneumonitis. Withont discussing the mode of its production as a rule in cases in which cavity does not exist, I will offer an explanation of its occurrence in this particular case. It will be seen by reference to the observation, that the second rib on the left side was remarkably yielding to pressure. The solid tuberculous mass behind the bronchus furnished a point of resistance, or, as it were, a fulcrum. On percussion, the ribs and the lung anteriorly to the bronchus yield readily, and the tuberculous deposit formed a kind of anvil causing the air in the bronchi to be expelled in such a way, and with sufficient force to yield the cracked pot sound. Of the existence of this sound in the case there was no doubt. While the patient was in the hospital, it was frequently demonstrated by myself and others as a good illustration of the sign.*

I can only account for the fact that the pitch at the summit of the left chest appeared lower than over the mammary region by snpposing that the presence of the small tuberculous masses in the lower lobe on this side, exalted the pitch more than the deposits in the upper lobe. Why this should be so it is not easy to understand. It is possible that the comparison, in these situations, was not made with sufficient care.

---

* Since this was written, a child admitted into the hospital, with affection of the mesenteric glands, reduced to extreme emaciation, but without cough or other symptoms of pulmonary affection, presents a very distinct *bruit de pot fele* on the left side.

This case furnished an interesting illustration of the effect of a moderate tuberculous mass, surrounded by vesicles, in modifying the pitch of resonance on percussion. The pitch of sound on percussion, in the left infra-clavicular region, was distinctly raised.

### 4 *Arrested Tubercle.*

*Obs.* 1.—Jan. 27. Martin Welch. Hospital patient. This patient presented, a year ago, the evidence, physical and rational, of small tuberculous deposit. The disease, in the mean time, has apparently made no progress. He remains in the hospital as a porter. Some cough and moderate expectoration continue. He has not lost in weight during the year, and his aspect is not notably morbid.

On auscultation, the respiration at the summit of the right side is relatively feeble, and the pitch is elevated. There is no sound of expiration, nor does the respiration present any abnormal characters, save feebleness and elevation of pitch.

On percussion, after obtaining the foregoing results, I find slight relative dulness in the right infra-clavicular region.

*Obs.* 2.—March 4. The subject of this observation is a professional friend of the writer. In 1844 he suffered for several consecutive months from cough with moderate expectoration. Hæmoptysis occurred during this period once. His weight was considerably diminished. He gradually recovered without medical treatment, and with the exception of a cough, which lasted for two months in 1849, he has enjoyed good health, being entirely free from any symptoms of pulmonary disease.

There is distinct elevation of the pitch of resonance on percussion at the summit of the chest on the left side. The respiration in this situation is slightly elevated in pitch, and the vesicular quality is somewhat less developed than on the right side.

Slight vocal resonance exists on both sides, in about an equal degree.

*Remarks.*—The variations in resonance and respiration being on the left side, are, on this account, the more significant of changes, probably due to arrested tubercle.

